# Practice Variation in Use of Neuroimaging Among Infants With Concern for Abuse Treated in Children’s Hospitals

**DOI:** 10.1001/jamanetworkopen.2022.5005

**Published:** 2022-04-20

**Authors:** M. Katherine Henry, Samantha Schilling, Justine Shults, Chris Feudtner, Hannah Katcoff, Teniola I. Egbe, Mitchell A. Johnson, Savvas Andronikou, Joanne N. Wood

**Affiliations:** 1 Safe Place: Center for Child Protection and Health, Division of General Pediatrics, Children’s Hospital of Philadelphia, Philadelphia, Pennsylvania; 2 Center for Pediatric Clinical Effectiveness, Children’s Hospital of Philadelphia, Philadelphia, Pennsylvania; 3 Department of Pediatrics, Perelman School of Medicine at the University of Pennsylvania, Philadelphia; 4 Department of Radiology, Children’s Hospital of Philadelphia, Philadelphia, Pennsylvania; 5 Division of General Pediatrics and Adolescent Medicine, University of North Carolina at Chapel Hill School of Medicine, Chapel Hill; 6 Division of Biostatistics, Department of Biostatistics, Epidemiology and Informatics, University of Pennsylvania Perelman School of Medicine, Philadelphia; 7 Division of General Pediatrics, Department of Pediatrics, Perelman School of Medicine at the University of Pennsylvania, Philadelphia; 8 Department of Medical Ethics, Children's Hospital of Philadelphia; 9 Data Science and Biostatistics Unit, Department of Biomedical and Health Informatics, Children’s Hospital of Philadelphia, Philadelphia, Pennsylvania; 10 Division of Orthopaedics, Children’s Hospital of Philadelphia, Philadelphia, Pennsylvania; 11 Perelman School of Medicine at the University of Pennsylvania, Philadelphia; 12 Policy Lab, Children’s Hospital of Philadelphia, Philadelphia, Pennsylvania

## Abstract

**Importance:**

Infants who appear neurologically well and have fractures concerning for abuse are at increased risk for clinically occult head injuries. Evidence of excess variation in neuroimaging practices when abuse is suspected may indicate opportunity for quality and safety improvement.

**Objective:**

To quantify neuroimaging practice variation across children’s hospitals among infants with fractures evaluated for abuse, with the hypothesis that hospitals would vary substantially in neuroimaging practices. As a secondary objective, factors associated with neuroimaging use were identified, with the hypothesis that age and factors associated with potential biases (ie, payer type and race or ethnicity) would be associated with neuroimaging use.

**Design, Setting, and Participants:**

This cross-sectional study included infants with a femur or humerus fracture or both undergoing abuse evaluation at 44 select US children’s hospitals in the Pediatric Health Information System (PHIS) from January 1, 2016, through March 30, 2020, including emergency department, observational, and inpatient encounters. Included infants were aged younger than 12 months with a femur or humerus fracture or both without overt signs or symptoms of head injury for whom a skeletal survey was performed. To focus on infants at increased risk for clinically occult head injuries, infants with billing codes suggestive of overt neurologic signs or symptoms were excluded. Multivariable logistic regression was used to investigate demographic, clinical, and temporal factors associated with use of neuroimaging. Marginal standardization was used to report adjusted percentages of infants undergoing neuroimaging by hospital and payer type. Data were analyzed from March 2021 through January 2022.

**Exposures:**

Covariates included age, sex, race and ethnicity, payer type, fracture type, presentation year, and hospital.

**Main Outcomes and Measures:**

Use of neuroimaging by CT or MRI.

**Results:**

Of 2585 infants with humerus or femur fracture or both undergoing evaluations for possible child abuse, there were 1408 (54.5%) male infants, 1726 infants (66.8%) who were publicly insured, and 1549 infants (59.9%) who underwent neuroimaging. The median (IQR) age was 6.1 (3.2-8.3) months. There were 748 (28.9%) Black non-Hispanic infants, 426 (16.5%) Hispanic infants, 1148 (44.4%) White non-Hispanic infants. In multivariable analyses, younger age (eg, odds ratio [OR] for ages <3 months vs ages 9 to <12 months, 13.2; 95% CI, 9.54-18.2; *P* < .001), male sex (OR, 1.47; 95% CI, 1.22-1.78; *P* < .001), payer type (OR for public vs private insurance, 1.48; 95% CI, 1.18-1.85; *P* = .003), fracture type (OR for femur and humerus fracture vs isolated femur fracture, 5.36; 95% CI, 2.11-13.6; *P* = .002), and hospital (adjusted range in use of neuroimaging, 37.4% [95% CI 21.4%-53.5%] to 83.6% [95% CI 69.6%-97.5%]; *P* < .001) were associated with increased use of neuroimaging, but race and ethnicity were not. Publicly insured infants were more likely to undergo neuroimaging (62.0%; 95% CI, 60.0%-64.1%) than privately insured infants (55.1%; 95% CI, 51.8%-58.4%) (*P* = .001).

**Conclusions and Relevance:**

This study found that hospitals varied in neuroimaging practices among infants with concern for abuse. Apparent disparities in practice associated with insurance type suggest opportunities for quality, safety, and equity improvement.

## Key Points

**Question:**

Does use of neuroimaging in infants assessed for possible child abuse vary by hospital?

**Findings:**

In this cross-sectional study of 2585 infants with humerus or femur fractures or both undergoing evaluation for possible child abuse, neuroimaging was performed in 37.4% to 83.6% of infants across hospitals. Publicly insured infants were significantly more likely to undergo neuroimaging than privately insured infants.

**Meaning:**

This study found excess practice variation and apparent disparities despite adjustment for case mix and temporal trends, suggesting opportunities for quality and equity improvement.

## Teaser

This cross-sectional study investigates variation in use of neuroimaging at children’s hospitals among infants with humerus or femur fractures or both undergoing evaluations for possible child abuse.

## Introduction

Infants who appear neurologically well and have injuries that raise concern for physical abuse (eg, extremity fractures) may have additional injuries not detected on physical examination. Identification of a clinically occult injury can guide medical care and increase the diagnostic certainty that an infant was abused. Clinically occult injuries can include intracranial injuries (eg, subdural hematoma). Infants with intracranial injuries from abuse can appear neurologically well with subtle or nonspecific signs or symptoms, such as fussiness or vomiting, or may lack external signs or symptoms associated with intracranial injury. Neuroimaging with computed tomography (CT) or magnetic resonance imaging (MRI) is required to identify these injuries, given that ultrasonography is not sufficiently sensitive.^1^

The process of deciding whether to perform neuroimaging to evaluate for occult intracranial injury includes weighing the risks associated with missed injury against the risks associated with radiation and sedation exposure.^2,3,4^ In addition, neuroimaging requires additional resources and may prolong length of stay. While clear guidelines inform use of plain radiography (ie, skeletal surveys) to identify clinically occult fractures,^1,5,6^ current guidelines are limited regarding use of screening neuroimaging to detect clinically occult head injuries in infants who appear neurologically well in the setting of suspected abuse. The American Academy of Pediatrics (AAP) recommends neuroimaging with MRI or CT to screen for occult head injuries in infants with “suspicious bruising”^5^ and that neuroimaging be “considered” among infants with “skeletal injuries associated with shaking or impact”^1^ and for infants with a “fracture suspicious for abuse”^7^ but without further clarification of the specific fractures.

Given the importance of detecting occult intracranial injuries, complexity of the decision to perform neuroimaging, and lack of clear practice guidelines, overassessment and underassessment may be occurring. If evidence of excess variation in neuroimaging practices when abuse is suspected were found, this would support the need for quality and safety improvement.^8,9^ Furthermore, there are known racial and ethnic and socioeconomic disparities in use of skeletal surveys to identify clinically occult fractures, suggesting bias in considering abuse.^10,11,12^ Whether biases continue after the initial decision to evaluate with a skeletal survey is unknown, but such continuation would further support the need for standardization of care. Once abuse is suspected, there may be subsequent biases in the degree of evaluation and willingness to consider other injuries, such as intracranial hemorrhage.

To evaluate these concerns, we designed a multicenter cross-sectional study of neuroimaging practices in emergency and inpatient care settings across children’s hospitals, limited to infants with a humerus or femur fracture or both. We did so because of the relatively high rate of diagnosed abuse in infants with these fractures compared with infants with other extremity fractures and because of recommendations that infants with these specific fractures receive evaluation for physical abuse.^7,13,14,15,16,17,18^ This restriction of the study population to patients with specific high-risk injuries may, if anything, have been associated with decreased evidence of bias and practice variation. Based on prior literature finding increased rates of skeletal surveys among Black infants and publicly insured infants, we hypothesized that these associations would extend to neuroimaging decisions.^10,11,12^ We also hypothesized that younger infants would be more likely to be imaged because they may be considered at increased risk for occult intracranial injury. Finally, we hypothesized that hospital-level variation would persist in neuroimaging use after adjustment for patient-level case mix and temporal trends.

## Methods

We performed a cross-sectional study of neuroimaging use among infants with humerus or femur fractures in the Pediatric Health Information System (PHIS), a large administrative database of more than 40 US children’s hospitals.^19^ The Children's Hospital of Philadelphia institutional review board determined that our study met exemption criteria per 45 CFR §46.104(d) 4(iii), which applies to secondary research for which consent is not required. This study follows Strengthening the Reporting of Observational Studies in Epidemiology (STROBE) reporting guideline for cross-sectional studies.

### Overview

We first identified a population of infants with femur and/or humerus fractures who underwent evaluations for physical abuse as evidenced by performance of a skeletal survey. We excluded infants with billing codes suggestive of neurological signs or symptoms for which neuroimaging would likely be clinically indicated. We used multivariable logistic regression to investigate factors associated with performing neuroimaging, and we describe variation in neuroimaging use across hospitals, with adjustment for case mix and temporal trends.

### Study Population

#### Inclusion

We included infants aged younger than 12 months with emergency department (ED) visits, admissions to observation status, or inpatient admissions with an *International Statistical Classification of Diseases, Tenth Revision, Clinical Modification *(*ICD-10-CM*) diagnosis code for femur fracture or humerus fracture or both (eAppendix in the Supplement). We focused on these fracture types given that they would be likely to prompt consideration of abuse in this age group and have been used to study imaging practices in abuse.^20,21,22^ Infants were included from January 1, 2016, through March 31, 2020. The AAP has clear guidance that a skeletal survey should be performed when there is concern for abuse in children younger than age 2 years.^1,5^ To focus on infants with concern for abuse, we limited our population to infants with Clinical Transaction Classification (CTC) codes indicating skeletal survey performance.

#### Exclusion

We excluded birth-related admissions and infants in motor vehicle collisions given that concern for physical abuse would be low in these populations. We also excluded infants with predisposition to fracture, defined as osteogenesis imperfecta or rickets, because the concern for abuse may have been lower and associated with neuroimaging decisions among these infants. To focus on neuroimaging for detection of clinically occult intracranial injuries, we attempted to exclude infants with potentially clinically apparent central nervous system conditions or head trauma who would have required neuroimaging based on signs and symptoms. First, we excluded infants with *ICD-10-CM* codes for epilepsy, posttraumatic seizure, macrocephaly, scalp hematoma, skull fracture, or intracranial injury associated with loss of consciousness. Second, we excluded infants with an *ICD-10-Procedure Coding System* code for neurosurgical intervention on day 0 or 1 of hospitalization. Third, we excluded infants whose neuroimaging was performed prior to the skeletal survey given that these infants likely presented with signs of intracranial injury. PHIS reports resource use by day of hospitalization, so infants with skeletal survey and neuroimaging on the same day were not excluded. We excluded infants transferred from other institutions given that these infants may have undergone imaging prior to transfer that would not be captured in our sample. Among children with multiple presentations, we included the index presentation or first presentation with a skeletal survey. We excluded infants presenting to hospitals that evaluated fewer than 5 infants meeting our inclusion and exclusion criteria.

### Outcome

Our primary outcome was use of neuroimaging (CT or MRI). We also explored the presence of intracranial injury as a descriptive outcome, defined broadly as an intracranial injury or hemorrhage from traumatic or nontraumatic causes (eAppendix in the Supplement).

### Covariates

Covariates included payer type, combined race and ethnicity, age category (ie, ages 0 to <3, 3 to <6, 6 to <9, and 9 to <12 months), sex, year of presentation, fracture type (ie, humerus, femur, or both), and hospital. In the PHIS database, race and ethnicity are coded separately; categories in the database are American Indian, Asian, Black, White, other, and missing for race and Hispanic, not Hispanic, and unknown for ethnicity. For race, PHIS also includes Native Hawaiian or Pacific Islander, but none were present in our study population. We assessed race to test our hypothesis that race may be associated with neuroimaging decisions. Because 147 of 287 infants (51.2%) in our population with race coded as other were coded as Hispanic ethnicity, we merged race and ethnicity into a single variable, as has been done in prior literature assessing disparities in PHIS.^10,23,24^ We recognize that race and ethnicity are distinct and that the resulting categories may not align with how families identify. The final race and ethnicity categories were American Indian non-Hispanic, Asian non-Hispanic, Black non-Hispanic, Hispanic, White non-Hispanic, and other or unknown non-Hispanic. We categorized payer type as private (ie, commercial or military), public (ie, Medicaid, other government, or Children's Health Insurance Program [CHIP]), or other or unknown (ie, missing, self-pay, charity, other payer, or cases in which the hospital did not bill).

### Quality Checks

#### Single-Center Validation

We used PHIS data from our hospital to identify 140 infants aged younger than 12 months who had an ED, observation, or inpatient encounter with a femur or humerus fracture or both within the study time frame not related to birth hospitalizations, motor vehicle collisions, or transfers, which were excluded via PHIS codes. After reviewing infants’ electronic health records, we calculated the sensitivity and specificity of our CTC definitions of skeletal survey and neuroimaging for performance of these modalities at our hospital or another medical center given that identification of transfers in PHIS may be imperfect. Our skeletal survey definition was 100% sensitive and 100% specific for skeletal survey performance at our hospital and 88.8% sensitive and 100% specific for performance at our hospital or another institution. Our CTC neuroimaging definition was 100% sensitive and 100% specific for neuroimaging performed at our hospital and 93.5% sensitive and 100% specific for performance at our hospital or another institution.

### Statistical Analysis

All analyses were performed in Stata statistical software version 16.1 (StataCorp) with 2-sided tests of hypotheses. We first performed descriptive statistics, reporting frequencies and proportions. We assessed for associations of age, sex, fracture type, year, payer type, and race and ethnicity with neuroimaging use in unadjusted χ^2^ analyses or Fisher exact test when cell size was less than 5. We then used multivariable logistic regression to estimate the association of these factors with odds of neuroimaging use. We a priori elected to include age, sex, fracture type, year, payer type, race and ethnicity, and hospital in our model. The significance level was set α = .05. We repeated our model, restricting the population to infants ages less than 6 months and separately to infants ages 6 to 12 months.

We report odds ratios (ORs) and the estimated probability of neuroimaging for publicly vs privately insured children and at each hospital.^25,26^ The estimated probability is based on marginal standardization,^20^ calculated by treating all individuals in this population as if they were publicly insured; we performed the same calculation for all individuals while treating them as if they were privately insured.^25^ The calculation across hospitals assumed that all infants in the sample presented to each individual hospital, calculating the mean estimated probability of neuroimaging at each hospital.^20^ We multiplied the estimated probabilities by 100 and describe these as adjusted percentages for ease of interpretation. We used CIs from the Stata margins command after logistic regression. CIs less than 0 or greater than 100 were clipped to 0.01% and 99.9%, respectively. Data were analyzed from March 2021 through January 2022.

## Results

### Study Population

**Figure 1.**
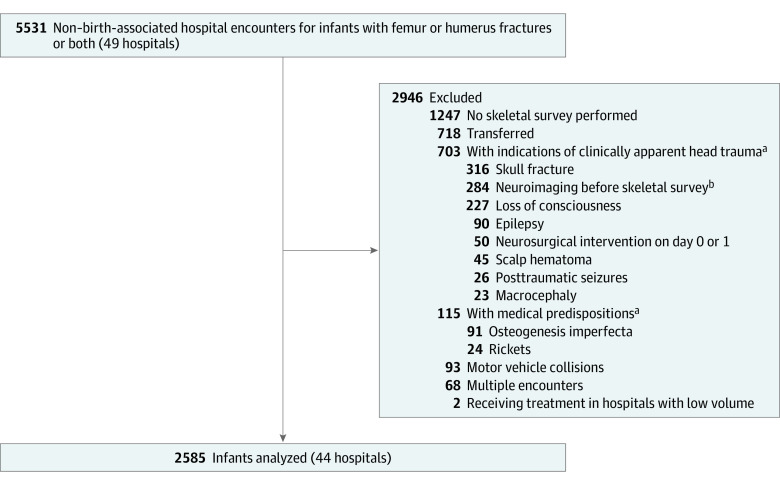
Population Flow Diagram ^a^Exclusion may be for more than 1 category. ^b^The Pediatric Health Information System provides use data by hospitalization day. Neuroimaging performed on the same day as the skeletal survey was included.

Our final study population included 2585 infants from 44 hospitals (Figure 1), who had a median (IQR) age of 6.1 (3.2-8.3) months and were predominately male (1408 [54.5%]) and publicly insured (1726 infants [66.8%]). There were 7 (0.3%) American Indian non-Hispanic infants, 54 (2.1%) Asian non-Hispanic infants, 748 (28.9%) Black non-Hispanic infants, 426 (16.5%) Hispanic infants, 1148 (44.4%) White non-Hispanic infants, and 202 (7.8%) infants with other or unknown race or ethnicity (Table 1). A total of 1549 infants (59.9%) underwent neuroimaging, of whom 48 infants (3.1%) had intracranial injury or hemorrhage.

**Table 1.** Study Population Characteristics

Characteristic	Infants, No. (%)^a^			*P* value^b^
	Total	Neuroimaging obtained		
		No	Yes	
Entire population	2585 (100)	1036 (40.1)	1549 (59.9)	NA
Age, mo				
0 to <3	621 (24.0)	85 (8.2)	536 (34.6)	<.001
3 to <6	651 (25.2)	157 (15.2)	494 (31.9)	
6 to <9	855 (33.1)	500 (48.3)	355 (22.9)	
9 to <12	458 (17.7)	294 (28.4)	164 (10.6)	
Sex				
Female	1177 (45.5)	549 (53.0)	628 (40.5)	<.001
Male	1408 (54.5)	487 (47.0)	921 (59.5)	
Insurance				
Private	719 (27.8)	360 (35.8)	359 (23.2)	<.001
Public	1726 (66.8)	621 (59.9)	1105 (71.3)	
Other or unknown	140 (5.4)	55 (5.3)	85 (5.5)	
Race and ethnicity^c^				
American Indian non-Hispanic	7 (0.3)	3 (0.3)	4 (0.3)	.01
Asian non-Hispanic	54 (2.1)	27 (2.6)	27 (1.7)	
Black non-Hispanic	748 (28.9)	261 (25.2)	487 (31.4)	
Hispanic	426 (16.5)	186 (18.0)	240 (15.5)	
White non-Hispanic	1148 (44.4)	480 (46.3)	668 (43.1)	
Other or unknown	202 (7.8)	79 (7.6)	123 (7.9)	
Extremity fracture				
Femur	1682 (65.1)	737 (71.1)	945 (61.0)	<.001
Humerus	841 (32.5)	293 (28.3)	548 (35.4)	
Both	62 (2.4)	6 (0.6)	56 (3.6)	
Year				
2016	576 (22.3)	234 (22.6)	342 (22.1)	.24
2017	593 (22.9)	252 (24.3)	341 (22.0)	
2018	654 (25.3)	265 (25.6)	389 (25.1)	
2019	635 (24.6)	244 (23.6)	391 (25.2)	
2020^d^	127 (4.9)	41 (4.0)	86 (5.6)	

Abbreviation: NA, not applicable.

^a^ Columns may not sum to 100 owing to rounding. Per convention, column percentages are presented, except in the first row, which includes row percentages. In the text, for ease of comparisons across different characteristics, row percentages are presented.

^b^ *P* value calculated from χ^2^ analyses, unless cell size was less than 5, in which case Fisher exact test was used.

^c^ In the Pediatric Health Information System, race and ethnicity are coded separately. Because 147 of 287 infants (51.2%) in the study population with race coded as other were coded as Hispanic ethnicity, race and ethnicity were merged into a single variable. Categories in the database for the study population are American Indian, Asian, Black, White, other, and missing for race and Hispanic, not Hispanic, and unknown for ethnicity.

^d^ Only quarter 1 of 2020 data included.

### Factors Associated With Neuroimaging Use

**Figure 2.**
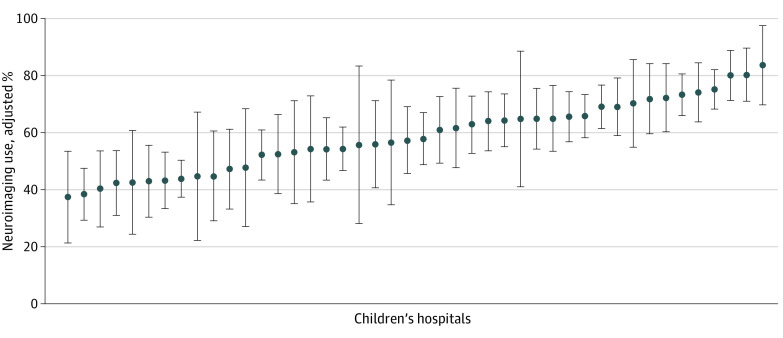
Variation in Neuroimaging Use Across 46 Children’s Hospitals Results were calculated by hospital using marginal standardization, with adjustment for payer type (ie, public, private, or other), race and ethnicity, age category (ie, ages 0 to <3, 3 to <6, 6 to <9, and 9 to <12 months), sex, year, fracture type (ie, humerus, femur, or both), and hospital in a logistic regression model. Children's hospitals are sorted from lowest to highest neuroimaging use.

In unadjusted analyses, race and ethnicity and payer type were associated with neuroimaging use. Neuroimaging was obtained in 4 of 7 American Indian non-Hispanic infants (57.1%), 27 of 54 Asian non-Hispanic infants (50.0%), 487 of 748 Black non-Hispanic infants (65.1%), 240 of 426 Hispanic infants (56.3%), 668 of 1148 White non-Hispanic infants (58.2%), and 123 of 202 infants with other or unknown race or ethnicity (60.9%) (*P* = .01). Neuroimaging was obtained among 359 of 719 infants with private insurance (49.9%), 1105 of 1726 infants with public insurance (64.0%), and 85 of 140 infants with other or unknown insurance (60.7%) (*P* < .001) (Table 1). After adjustment for age, sex, race and ethnicity, fracture type, and hospital (Figure 2 and Table 2), we found that publicly insured infants more commonly underwent neuroimaging (62.0%; 95% CI, 60.0%-64.1%) than privately insured infants (55.1%; 95% CI, 51.8%-58.4%) (*P* = .001) (Table 2). In this setting, we assessed whether yield of neuroimaging was increased among privately vs publicly insured infants. There was no association between payer type and detection of an intracranial injury; among infants who underwent neuroimaging, 6 of 359 privately insured infants (1.7%) vs 41 of 1105 publicly insured infants (3.7%) had an intracranial hemorrhage or injury (*P* = .10). There was no association between race and ethnicity and neuroimaging use in adjusted analyses.

**Table 2.** Factors Associated With Neuroimaging Use

Factor^a^	OR (95% CI)	*P* value^b^
Age, mo		
9 to <12	1 [Reference]	<.001
0 to <3	13.2 (9.54-18.2)	
3 to <6	6.33 (4.76-8.42)	
6 to <9	1.33 (1.03-1.71)	
Sex		
Female	1 [Reference]	<.001
Male	1.47 (1.22-1.78)	
Insurance		
Private	1 [Reference]	.003
Public	1.48 (1.18-1.85)	
Other or unknown	1.29 (0.82-2.05)	
Race and ethnicity^c^		
American Indian non-Hispanic	0.62 (0.11-3.33)	.48
Asian non-Hispanic	0.92 (0.48-1.76)	
Black non-Hispanic	1.23 (0.96-1.56)	
Hispanic	0.98 (0.73-1.33)	
White non-Hispanic	1 [Reference]	
Other or unknown	1.23 (0.85-1.77)	
Extremity fracture		
Femur	1 [Reference]	.002
Humerus	1.05 (0.86-1.29)	
Both	5.36 (2.11-13.6)	
Year		
2016	1 [Reference]	.26
2017	0.93 (0.71-1.23)	
2018	1.10 (0.84-1.45)	
2019	1.11 (0.85-1.47)	
2020	1.54 (0.95-2.49)	

Abbreviation: OR, odds ratio.

^a^ The association of hospital with neuroimaging use is presented as adjusted percentages in Figure 2.

^b^ Wald test.

^c^ In the Pediatric Health Information System, race and ethnicity are coded separately. Because 147 of 287 infants (51.2%) in the study population with race coded as other were coded as Hispanic ethnicity, race and ethnicity were merged into a single variable. Categories in the database for the study population are American Indian, Asian, Black, White, other, and missing for race and Hispanic, not Hispanic, and unknown for ethnicity.

In unadjusted analyses, younger age (eg, 536 of 621 infants ages 0 to <3 months [86.3%] vs 164 of 458 infants ages 9 to <12 months [35.8%] with neuroimaging; *P* < .001) and male sex (921 of 1408 male infants [65.4%] vs 628 of 1177 female infants with neuroimaging [53.4%]; *P* = <.001) were associated with increased neuroimaging use, and having a femur and humerus fracture simultaneously (56 of 62 infants [90.3%] vs 548 of 841 infants with a humerus fracture [65.2%] and 945 of 1682 infants with a femur fracture [56.2%]; *P* < .001) was associated with increased neuroimaging use (Table 1). In adjusted analyses (Table 2), young age (eg, OR for age <3 months vs 9 to <12 months, 13.2; 95% CI, 9.54-18.2; *P* < .001), male sex (OR 1.47; 95% CI, 1.22-1.78; *P* < .001), payer type (OR for public vs private insurance, 1.48; 95% CI, 1.18-1.85; *P* = .003), and having a femur and humerus fracture (OR vs isolated femur fracture, 5.36; 95% CI, 2.11-13.6; *P* = .002) were associated with higher odds of neuroimaging use.

### Hospital-Level Practice Variation in Neuroimaging

In unadjusted analyses, the percentage of infants undergoing neuroimaging by hospital ranged from 5 of 16 infants (31.3%) to 63 of 76 infants (82.9%). After adjustment for age, sex, fracture, payer type, race and ethnicity, and year, substantial hospital variation in neuroimaging practices remained, ranging from 37.4% (95% CI 21.4%-53.5%) to 83.6% (95% CI 69.6%-97.5%) (Figure 2). There was a statistically significant difference in neuroimaging use across hospitals (*P* < .001).

### Age-Limited Models

When limited to infants ages younger than 6 months, the adjusted percentage undergoing neuroimaging ranged from 48.7% (95% CI, 24.4 %-73.0%) to 96.9% (95% CI, 90.9%-99.9%) across hospitals (*P* = .01). Among infants ages 6 to 12 months, the adjusted percentage of neuroimaging across hospitals ranged from 4.1% (95% C1, 0.01%-13.4%) to 92.6% (95% CI, 78.8%-99.9%) (*P* < .001).

Among infants ages less than 6 months, the adjusted percentage of publicly insured infants undergoing neuroimaging was 81.6% (95% CI, 79.0%-84.1%) compared with 76.3% (95% CI 71.4-81.2) among privately insured infants (*P* = .08). In infants ages 6 to 12 months, publicly insured infants (42.7%; 95% CI 39.6%-45.8%) were more likely to undergo neuroimaging than privately insured infants (33.5%; 95% CI 29.0-38.0) (*P* = .003).

### Hospital Reporting Sensitivity Analysis

Hospital reporting of data to PHIS differed over time, most notably in admissions to observation status. We therefore performed a sensitivity analysis excluding admissions to observation status. This did not change the magnitude or significance of our model’s findings.

## Discussion

In our cross-sectional study of neuroimaging use and variation among infants with humerus and femur fractures or both evaluated for abuse, we found that performance of neuroimaging varied substantially across hospitals, despite adjustment for case mix and temporal trends. Publicly insured infants underwent neuroimaging more often compared with their privately insured counterparts. These findings suggest opportunities for quality, safety, and equity improvement.

Our results align with and add to prior studies of hospital variation in abuse evaluations. Prior work found variation in skeletal survey performance and abuse diagnoses in injured infants.^20,21^ Less is known about variation in neuroimaging practices. In one study,^21^ investigators described the medical evaluation for young children with a variety of injuries within PHIS from 2004 through 2011. Overall in that study, 63.8% of infants ages younger than 12 months with a femur or humerus fracture underwent neuroimaging, but neuroimaging use by hospital ranged from 43.3% to 84.7%.^21^ In addition to our use of contemporary data, our population and methods differ in several critical ways from previous studies: (1) all infants in our study had concern for abuse (defined by skeletal survey performance), (2) efforts were made to exclude infants who may have had clinically indicated neuroimaging, and (3) we adjusted for case mix and temporal trends. Despite these key differences, we nonetheless similarly found that 59.9% of infants underwent neuroimaging, with neuroimaging use ranging from 37.4% to 83.6% across hospitals.

Our results add to prior literature in finding that disparities in abuse-related imaging evaluations in young children with injuries extend beyond the initial decision to evaluate for abuse by obtaining a skeletal survey. Prior work has found associations of race and socioeconomic status (SES) with skeletal survey use.^10,11,12^ In our study, race and ethnicity and payer type were associated with neuroimaging use in unadjusted χ^2^ analyses. After adjustment for multiple factors, the association between public insurance and neuroimaging use remained, while there was no association for race and ethnicity. Interpreting payer type as one proxy for SES,^27^ our results suggest disproportionate neuroimaging of infants with concern for abuse among families with lower SES. These results align with prior findings that children with fractures among families with lower SES are more likely to be evaluated with a skeletal survey and reported to child protective services.^12^ While poverty is a risk factor associated with abuse,^28^ SES should not be considered when making the decision to evaluate for occult head trauma in an infant with a serious injury when abuse is being assessed. These results suggest that once the decision is made to assess for abuse with a skeletal survey, there are sequential biases in decision-making.

In unadjusted and adjusted analyses, we found that young infants were more likely to undergo neuroimaging. These findings fit with the clinical practice of child abuse pediatricians who have a lower threshold to obtain neuroimaging in infants aged less than 6 months.^29^ Some studies,^30,31^ but not all studies,^32,33^ have found an increased percent positivity of neuroimaging studies for occult head injuries in younger infants undergoing evaluations for abuse compared with older infants and children. In fact, we found that hospitals varied more widely in the decision to image older infants compared with those aged younger than 6 months. The differences in the percent probability of neuroimaging we found in the setting of public vs private insurance remained among older infants but abated when limited to those aged younger than 6 months.

Our results should be interpreted within the context of the evolving data on yield of neuroimaging in detection of occult head trauma in suspected abuse. In the early 2000s, Rubin and colleagues’ landmark study^30^ sparked increasing recognition of the risk of occult head injuries in asymptomatic infants and young children undergoing abuse evaluations. Early studies^30,34^ suggested neuroimaging yields of 29% to 37%. In the past 2 decades, researchers have continued to assess screening neuroimaging yield (ie, percent positivity for occult head injury) in observational studies. As imaging practices and definitions of occult head injuries have evolved over time, multiple recent studies suggest yields of less than 10%^31,32,35^ or even less than 1%.^36^ In our administrative database study, neuroimaging identified intracranial injury or hemorrhage in 3.1% of individuals, similar to the yield identified in a prior retrospective chart review in the setting of extremity fractures.^37^

Our data suggest that with current neuroimaging guidance, clinicians across different children’s hospitals took disparate approaches to neuroimaging. In addition, our study findings suggest that biases based on patient demographics may be associated with imaging decisions. Guidance regarding neuroimaging has not undergone recent revision. This guidance encourages clinicians to “consider” neuroimaging in certain situations, which are not well-defined and thus interpreted differently.^1,7^ The previously listed studies with lower neuroimaging yields in combination with our findings suggest opportunities to develop an evidence-based, targeted neuroimaging approach with concrete guidance to reduce biases and further standardize care.

### Limitations

Use of PHIS allowed us to study a large population of infants with concern for inflicted extremity fractures, but this administrative data set comes with several limitations. First, PHIS provides diagnoses at the end of the clinical encounter. We were unable to discern presenting symptoms or presenting injuries. Infants could have had additional injuries or fractures that prompted or increased concern for abuse. We made efforts to exclude children who presented with overt neurological signs or symptoms, including infants undergoing neurosurgical procedure early in their hospital course. While we excluded infants with codes for other findings, such as seizures, we cannot be sure that seizures were observed on presentation. Second, we were not able to adjust for all factors that clinicians may weigh when making neuroimaging decisions, such as the plausibility of the history provided to explain the fracture or developmental abilities of the child. Third, many injured children receive care at institutions other than children’s hospitals.^38^ Our findings may not be generalizable to care settings beyond children’s hospitals.

## Conclusions

This study found that children’s hospitals vary in neuroimaging practices among infants with concern for inflicted extremity fractures. In unadjusted and adjusted analyses, publicly insured infants more frequently underwent neuroimaging than their privately insured peers. Our results suggest opportunities to improve the quality, safety, and equity of neuroimaging practices among infants undergoing evaluations for suspected physical abuse.

## Notes

Supplement. **eAppendix.** Study Code Definitions
