# Supplementary material for: Practice Variation in Use of Neuroimaging Among Infants With Concern for Abuse Treated in Children’s Hospitals
Source: JAMA Netw Open. 2022 Apr 20;5(4):e225005. doi: 10.1001/jamanetworkopen.2022.5005 (PMC9021910; doi:10.1001/jamanetworkopen.2022.5005)
Supplement: Supplement. — eAppendix. Study Code Definitions [file jamanetwopen-e225005-s001.pdf]

Supplemental Online Content

Henry MK, Schilling S, Shults J, et al. Practice variation in use of neuroimaging among infants with concern for abuse treated in children's hospitals. *JAMA Netw Open*. 2022;5(4):e225005. doi:10.1001/jamanetworkopen.2022.5005

eAppendix. Study Code Definitions

This supplemental material has been provided by the authors to give readers additional information about their work.

© 2022 Henry MK et al. *JAMA Network Open.*

eAppendix. Study Code Definitions

Description	Code Type	Specific Code(s)
**Inclusions**		
Humerus Fracture	ICD-10-CM	S42.2<br>S42.3<br>S42.4<br>(only subcodes ending A/B/C to capture only initial encounters rather than subsequent)
Femur Fractures	ICD-10-CM	S72.0<br>S72.1<br>S72.2<br>S72.3<br>S72.4<br>S72.8<br>S72.9<br>(only subcodes ending A/B/C)
Skeletal Survey	CTC	427811<br>427463
**Exclusions**		
Hospitalization associated with birth	ICD-10-CM	Z38 and subcodes
Motor Vehicle Collision	ICD-10-CM	V00-V99 (and subcodes)<br>Y92.41 (and subcodes)
Osteogenesis imperfecta	ICD-10-CM	Q78.0
Rickets	ICD-10-CM	E55.0, E64.3
Neurosurgical procedure on day 0 or 1	ICD-10-PCS	001-00X
Epilepsy	ICD-10-CM	G40 and subcodes
Post traumatic seizures	ICD-10-CM	R56.1
Macrocephaly	ICD-10-CM	Q75.3
Scalp hematoma	ICD-10-CM	S00.03XA
Skull fracture	ICD-10-CM	S02.0, S02.1, S02.8, S02.9 (only subcodes ending in A/B/C)
Loss of consciousness (LOC)	ICD-10-CM	All subcodes of S06 reporting LOC
**Outcomes**		
Neuroimaging (CT or MRI)	CTC	411051<br>411052<br>411152<br>417051<br>417052<br>470052<br>471051<br>471052

© 2022 Henry MK et al. *JAMA Network Open.*

		481052
Intracranial injury / bleed		S06.1-9, I62, T74.4, P10, T90.5

© 2022 Henry MK et al. *JAMA Network Open.*
